# Physical and Mechanical Effects of Silica Sand in Cement Mortars: Experimental and Statistical Modeling

**DOI:** 10.3390/ma16216861

**Published:** 2023-10-25

**Authors:** Abdellah Douadi, Kamel Hebbache, Mourad Boutlikht, Seifeddine Tabchouche, Cherif Belebchouche, Redha Hammouche, Giulia Del Serrone, Laura Moretti

**Affiliations:** 1 Civil Engineering Research Laboratory of Setif (LRGCS), Department of Civil Engineering, Ferhat Abbas University of Setif 1, Setif 19000, Algeria; 2 Materials and Durability of Construction Laboratory, Department of Civil Engineering, Faculty of Science and Technology, Frére Mentouri University of Constantine 1, Constantine 25000, Algeria; 3 Department of Civil, Constructional and Environmental Engineering, Sapienza University of Rome, Via Eudossiana 18, 00184 Rome, Italy; giulia.delserrone@uniroma1.it

**Keywords:** water absorption, compressive strength, silica sand, cement substitution

## Abstract

The environmental impacts of cement manufacturing are becoming a real-time issue that requires attention. This paper investigates the mechanical and physical properties of mortars with finely ground sand as a substitute for cement. The experimental program consisted of three silica sands with a Blaine Specific Surface (BSS) area of 459 m^2^/kg, 497 m^2^/kg, and 543 m^2^/kg and four substitution ratios of 10%, 20%, 30%, and 40%. A total of 12 mixtures have been prepared and tested for comparison to the reference mortar. The pozzolanic effect of the sand was evaluated using thermogravimetric analysis (TGA). The results revealed that the fineness variation from 459 m^2^/kg to 543 m^2^/kg resulted in an increase of 20% and 30% in water absorption and compressive strength, respectively. However, increasing the substitution ratio from 10% to 40% led to a 40% decrease in mechanical strength and a 25% increase in water absorption. The statistical analysis of the results demonstrated that both factors under study influenced compressive strength and water absorption. The ANalysis of VAriance (ANOVA) confirmed that the proposed regression equations predict the experimental results. Further studies will investigate both the technical and environmental performances of cement mortars with finely ground silica sand.

## 1. Introduction

The cement industry contributes to greenhouse gas emissions, particularly carbon dioxide [1,2,3,4]. The main contribution is due to the production of clinker, which is the main component of cement. It involves a chemical reaction where carbonates (e.g., CaCO_3_) decompose into oxides (e.g., CaO) and CO_2_ through heat application [3]. This reaction requires fuel combustion to feed rotary kilns [5]. Accordingly, research for low-impact cement composed of alternative raw materials became a concern to solve this issue. De Medeiros et al. [6] tested the effects of fly ash on cement paste durability. The results indicated a decrease in the alkali contribution and compressive strength at 28 days, while there was an increase in the carbonation ratio. Patil et al. [7] conducted an experimental study by partially substituting cement with fly ash and bagasse ash in different ratios ranging from 0% to 30%. The results of the Frattini test showed that cement with 20% fly ash and 10% bagasse ash was pozzolanically active. The activity index of strength at 7 and 28 days exceeds the recommended value of 0.75. Hamidi et al. [8] investigated the andesite rock as a partial substitute for cement. Andesite exhibited moderate pozzolanic activity, surpassing that of natural pozzolans. Tayeh et al. [9] tested a mortar containing glass powder and observed an up to 8% increase in compressive strength for mixtures containing 20% glass powder; these findings comply with [10]. Furthermore, mortars containing glass powder exhibited good performance in external exposure to MgSO_4_ solution. Ramezanianpour et al. [11] observed that natural pozzolans and nanosilica reduce mortars’ workability due to their morphology and specific surface area higher than that of Portland cement. Cheah et al. [12] tested granulated blast furnace slag and ground granulated blast furnace slag containing calcium carbonate as partial substitutes for cement. A comparison between cement substitution with quartz and ash revealed that cement pastes containing quartz powder exhibited higher early compressive strength due to the physical filling of quartz particles [13]. Lin et al. [14] have reported that the quartz powder has both effects of dilution and crystal nucleation on cement hydration. Therefore, the incorporation of quartz powder does not affect the hydration product. Ma et al. [15] found a dilution effect of coral sand powder that delays the exothermic ratio of the cement system. Mortars containing fly ash and crushed river sand were more resistant to chloride penetration than the reference mortar [16]. This improvement is due to the filling effect, increased nucleation sites, a decrease in Ca(OH)_2_ content, and the presence of fine fly ash particles. The Kubuqi desert sand powder (North China) improved the early hydration of cement through nucleation, filling, and dilution [17]. Indeed, the sand particles enhanced the cement hydration, and their quartz and calcite contributed to the precipitation of early hydration products.

Other studies focused on supplementary materials in cement production [18] to reduce costs from quarries [19], decrease CO_2_ emissions [18,20], and enhance construction materials [21]. In the last few years, the use of industrial or natural pozzolanic materials (e.g., blast furnace slag, fly ash, silica fume, kaolin, and pozzolana) has been significantly reduced due to [22,23] environmental pressures related to energy consumption, steel recycling, which reduced the production of waste materials to be recycled, and the depletion of natural deposits [24,25].

In this context, finding abundant and alternative pozzolanic materials equivalent to those in use is strategic. Silica sand is one of the most widely available materials around the world. It derives from natural sources and is composed of SiO_2_ [21]. Quartz exhibits physical interactions with the cement hydration process (e.g., cement grain dilution, cement hydrate nucleation, and space-filling [14,26]). Table 1 summarizes the results of studies on silica sand as a substitute or mineral addition to construction materials.

Despite its availability, few studies have investigated silica sand and proven a significant gap concerning the sand fineness effect on the mechanical and physical properties of binary mortars. Therefore, this study aims to predict the mechanical and physical behavior of mortars containing silica sand by varying its fineness and cement substitution ratio (SP).

## 2. Materials and Methods

In this study, the samples were prepared and manufactured with CEM I 52.5 N Portland cement (Schwenk, Ulm, Germany). The used cement has 3150 kg/m^3^ density and 342 m^2^/kg Blaine Specific Surface (BSS). Its chemical composition is in Table 2. The sand used as a substitute was quarried in the municipality of El Gor, Tlemcen province (north-west Algeria), and had a high quartz content (98.91% by weight in Table 2). It was dried and ground in a ball mill to achieve a fineness lower than that of the cement.

Three types of silica sand with 459 m^2^/kg, 497 m²/kg, and 543 m²/kg BSS (BSS1, BSS2, and BSS3, respectively) were investigated.

**Figure 1:**
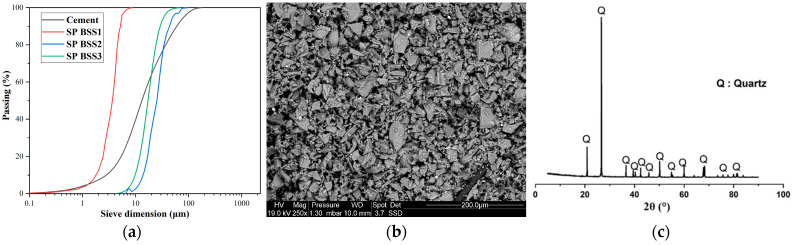
(**a**) Particle size distribution of cement and silica sand; (**b**) SEM image of silica sand; (**c**) X-ray diffraction of silica sand.

Figure 1a,b show the particle size distribution of cement and silica sand. The particle size analysis was compliant with [36] using a Mastersizer 2000 laser analyzer (Malvern Panalytical, Malvern, UK) and the scanning electron microscope “SEM” Philips/FEI XL 30S FEG Chatsworth, CA, USA. It is possible to observe an almost homogeneous size distribution for a range of sizes from 0 to 100 µm (Figure 1a). According to Figure 1b, grains of silica sand have an angular-shaped morphology. X-ray fluorescence spectroscopy was used to determine the chemical composition of the investigated materials, and a powder X-ray diffractometer identified the minerals in the samples. In Figure 1c, the X-ray diffraction pattern of silica sand confirms that quartz is the main mineral with a hexagonal structure.

This study compared four mixtures with 10%, 20%, 30%, and 40% cement substitution (M1, M2, M3, and M4, respectively) to the reference mixture (M0). Table 3 illustrates their chemical composition.

The hydraulic index (I) of M0 (i.e., 0.39 in Table 4) indicates it is neutral. It is the ratio of the acidic to the basic fraction of cement. The increasing substitution ratio of up to 40% allowed the transition of M0 to silica-rich mixtures (i.e., I > 1) (Table 4). Therefore, the hydraulic index has a direct correlation with the substitution ratio of crushed sand.

The ratios of binder/sand and water/binder were 0.33 and 0.5, respectively (Table 4). After mixing according to [37], each mixture was poured into 40 × 40 × 160 mm^3^ molds. Twenty-four hours after the initial set, the specimens were removed from the molds and stored in a water tank at room temperature until the test. A total of thirteen mixes were prepared and tested. Three samples were tested for each mortar.

Thermogravimetric analyses (TGA) quantified the non-evaporable water (NEW) and the portlandite percentage. Mortar samples were taken from crushed mixture cubes and immersed in acetone to halt hydration and prevent carbonation. After this step, the samples were carefully dried in a desiccator to eliminate residual moisture. Finally, the samples were ground and sieved through an 80 µm mesh sieve to obtain a homogeneous particle size distribution. Equation (1) allows NEW calculation [38]:

(1) $$NEW=\frac{W_{105{}^{\circ}}-W_{450{}^{\circ}}}{W_{450{}^{\circ}}}$$

Equation (2) gave the portlandite percentage (CH) [38]:

(2) $$CH=\left(W_{450{}^{\circ}}-W_{550{}^{\circ}}\right)\frac{M_{Ca{\left(OH\right)}_{2}}}{M_{H_{2}O}}+\left(W_{680{}^{\circ}}-W_{780{}^{\circ}}\right)\frac{M_{Ca{\left(OH\right)}_{2}}}{M_{CO_{2}}}$$

where $W_{105{}^{\circ}}$, $W_{450{}^{\circ}}$, $W_{550{}^{\circ}}$, $W_{680{}^{\circ}}$, and $W_{780{}^{\circ}}$ are the mass losses at 105 °C, 450 °C, 55 °C, 680 °C, and 780 °C, respectively; $M_{Ca{\left(OH\right)}_{2}}$, $M_{H_{2}O}$, and $M_{CO_{2}}$ are the molecular masses of portlandite, water, and carbon dioxide, respectively.

The flexural and compressive strength values at 7 and 28 curing days (i.e., R_f_7, R_f_28, CS7, and CS28) derive from Equations (3) and (4) [37], respectively:

(3) $$R_{f}=\frac{1.5\times F_{f}\times l}{b^{2}\times d}$$

(4) $$CS=\frac{F_{c}}{b^{2}}$$

where l is the distance between supports, $b^{2}$ is the cross-sectional area of the specimen, d is the specimen thickness, and $F_{f}$ and $F_{c}$ are the applied forces.

Immersion absorption tests were conducted to evaluate the durability of mortars [39,40]. After a curing process of 28 days, the samples were dried in an oven until their mass reached a constant value. Subsequently, they were immersed in a water tank for 24 h. Equation (5) allowed the calculation of the water absorption (WA) coefficient:

(5) $$WA=\frac{M_{2}-M_{1}}{M_{1}}\times 100$$

where $M_{1}$ and $M_{2}$ are the initial (i.e., after drying) and the final (i.e., after immersion) mass, respectively.

A rigorous statistical analysis investigated the influence of two independent variables (i.e., substitution ratios of 10%, 20%, 30%, and 40%, and Blaine fineness of 459 m^2^/kg, 497 m^2^/kg, and 543 m^2^/kg) on CS7, CS28, and WA. The authors used the JMP Pro 17 software, SAS for Universities Edition (SAS Institute Inc., Cary, NC, USA) [41] to obtain full factorial models (Equations (6)–(8)).

(6) $$\begin{array}{cc} CS7= & 37.25+9.31\times \left(\frac{\left(BSS\left(\frac{m^{2}}{\mathrm{kg}}\right)-501\right)}{42}\right)-10.89\times \left(\frac{\left(SP\left(\%\right)-25\right)}{15}\right)\\ & \;\;\;\;+\left(\left(\frac{\left(BSS\left(\frac{m^{2}}{\mathrm{kg}}\right)-501\right)}{42}\right)\times \left(\frac{\left(SP\left(\%\right)-25\right)}{15}\right)\times -2.78\;\right) \end{array}$$

(7) $$\begin{array}{cc} CS28= & 47.86+5.90\times \left(\frac{\left(BSS\left(\frac{m^{2}}{\mathrm{kg}}\right)-501\right)}{42}\right)-12.97\times \left(\frac{\left(SP\left(\%\right)-25\right)}{15}\right)\\ & \;\;\;\;+\left(\;\left(\frac{\left(BSS\left(\frac{m^{2}}{\mathrm{kg}}\right)-501\right)}{42}\right)\times \left(\frac{\left(SP\left(\%\right)-25\right)}{15}\right)\times -0.87\;\right) \end{array}$$

(8) $$\begin{array}{cc} WA= & 5.68+0.59\times \left(\frac{\left(BSS\left(\frac{m^{2}}{\mathrm{kg}}\right)-501\right)}{42}\right)+0.67\times \left(\frac{\left(SP\left(\%\right)-25\right)}{15}\right)\\ & \;\;\;\;+\left(\;\left(\frac{\left(BSS\left(\frac{m^{2}}{\mathrm{kg}}\right)-501\right)}{42}\right)\times \left(\frac{\left(SP\left(\%\right)-25\right)}{15}\right)\times -0.024\;\right) \end{array}$$

The ANalysis Of VAriance (ANOVA) evaluated the components’ contribution to the responses. The statistical significance of the models was assessed using the Fisher test distribution with a 95% confidence level [42]. The factors’ influence and their interaction were assessed using the Student’s *t*-test (Equation (9)).

(9) $$t=\frac{\bar{x}-\mu }{S_{\bar{x}}/\sqrt{n}}$$

where t is the test statistic, $\bar{x}$ is the sample mean, μ is the population mean, $S_{\bar{x}}$ is the sample standard deviation, and *n* is the sample size.

t is compared to a critical value $t_{crit}$ for a significance level α and degree of freedom (df=n−p) where p is the number of coefficients in the model. The critical value can be read from the Student’s t-distribution table [42]. If the absolute value of t is higher than $t_{crit}$, the effect of $a_{i}$ is significant; otherwise, it is not.

The compressive and flexural strengths at 7 and 28 days of the mortars have been investigated according to Equation (10) using the mechanical performance ratio (MPR) [43].

(10) MPR7days,  28days=4×CS7,28MiCS7,28M0+2×Rf7,28MiRf7,28M06×100

## 3. Results

Table 5 lists the mechanical and physical performances of the mortars according to Equations (3)–(5).

M0 and M4-BSS2 have been tested for 90 days. Their compressive strength values are 62 MPa and 39.6 MPa, and their flexural strength values are 8.46 MPa and 4.90 MPa, respectively. Therefore, the highest SP with 497 m^2^/kg silica sand implies a 40% decrease in mechanical strength after 90 curing days.

**Figure 2:**
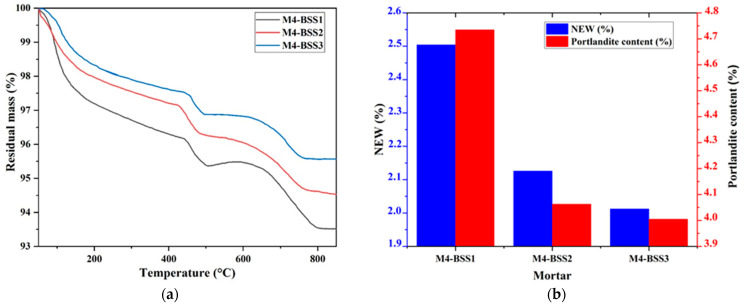
TGA of M4. (**a**) residual mass curves; (**b**) hydration degree and amount of portlandite at 1-day curing.

Figure 2 presents the TGA results of M4 after 1-day curing by varying BSS. Figure 2a has residual mass curves, and Figure 2b shows NEW and portlandite content.

Figure 2 shows a slight mass loss of M4 caused by silica sand fine particles at an early age. However, the variation in NEW and Ca(OH)_2_ content concerning BSS is not significant due to the nucleation phenomenon [44,45].

**Figure 3:**
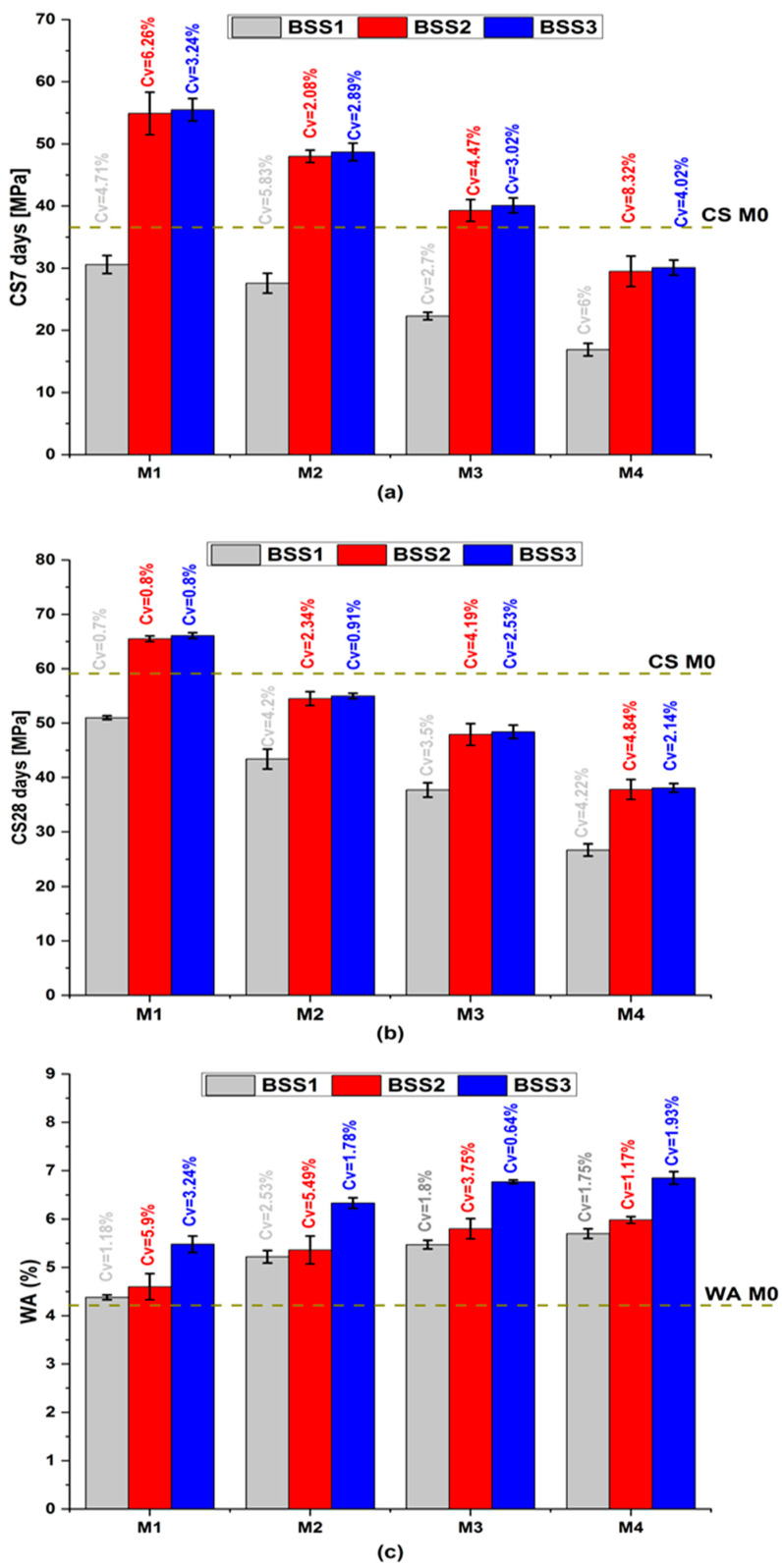
Mortar properties. (**a**) compressive strength at 7 days; (**b**) compressive strength at 28 days; (**c**) water absorption.

Figure 3 shows the average mortar properties of CS7, CS28, and WA with their coefficient of variation (Cv). In Figure 3a,b, the compressive strength exhibits a comparable qualitative trend with varying BSS [46,47]. At the same age, CS decreases with the increase in cement substitution and the resulting increase in water-to-cement ratio (W/C) [48]. The decrease in cement content increases the hydraulic index (Table 4), reduces hydration products that confer mechanical strength to mortars [49,50], and causes the formation of large capillary pores. On the other hand, CS increases with an increase in silica sand BSS. This behavior is due to the sand’s fineness exceeding that of cement (Figure 1a). The BSS2 and BSS3 values ensure that M1 to M3 CS7 and M1 CS28 are higher than M0. The pozzolanic effect of the silica sand justifies its mechanical performance because it contributes to the formation of the amorphous and dense calcium silicate hydrate gel [51,52]. Whatever the cement substitution ratio, the CS7 and CS28 values of mortars containing BSS2 sand are close to those containing BSS3 sand (red and blue bars in Figure 3a,b, respectively). It demonstrates the benefits of silica sand as pozzolanic and a filler material [49,53,54]. Therefore, BSS2 sand is recommended for binary mortars because it balances mechanical and environmental goals concerning high CS values and low grinding energy.

In Figure 3c, the increase in sand BSS implies an increase in WA because the sand particles are finer than those of cement (Table 1) and require more water. It causes a decrease in the cement hydrate volume and an increase in porosity and water absorption. The simultaneous increase in BSS and substitution ratio causes an increase in WA from 4.2% (M0) to 6.8% (M4-BSS3). The observed trend is attributed to the angular shape of silica sand grains, making granular stacking a bit challenging. However, the WA of binary mortars remains below 10% and ensures durability in aggressive environments [55].

**Figure 4:**
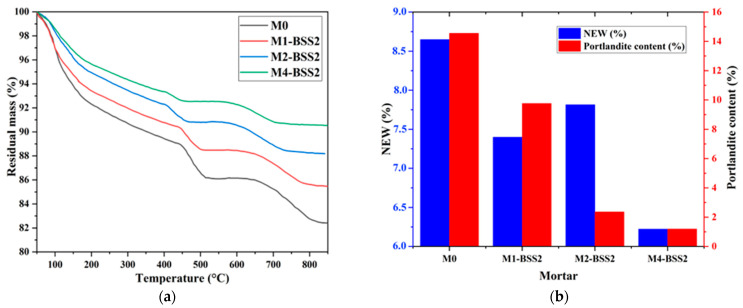
TGA of a mixture with different silica sand substitution ratios. (**a**) residual mass curves; (**b**) NEW and portlandite content at 7 days.

Figure 4a shows the residual masses of M0 and binary mortars containing BSS2 sand by varying the cement substitution ratio. Figure 4b represents the NEW and portlandite content of M0 and binary mortars containing BSS2 sand after 7-day curing.

The results revealed an inversely proportional relationship between the sand substitution ratio and NEW, which decreased from 8.65% to 6.22% from M0 to M4-BSS2, respectively (Figure 4b). According to [56], the dilution effect occurring when a pozzolanic additive substitutes the cement can explain that. The pozzolanic behavior of silica sand causes the partial consumption of calcium hydroxide ions produced during the cement hydration, resulting in a M0 to M4 decrease in Ca(OH)_2_ from 14.55% to 1.19%, respectively (Figure 4b).

Table 6 compares M0 and M4-BSS2 NEW and CH values at 7 and 90 days.

According to Table 6, NEW increases with time. M4-BSS2 is 28% and 18% lower than M0 after 7 and 90 days of hydration, respectively. A dilution effect [57,58] can explain this trend. Furthermore, the effective contribution of sand to hydration occurs in the long term. Over time, an increase in Ca(OH)_2_ in both samples reveals the formation of hydrated calcium silicate compounds due to hydration reactions [8,17]. In M0, CH contents increase with sample age because hydration leads to the formation of hydrated solids. The quantity of CH in M4-BSS2 is lower than in M0 due to dilution and pozzolanic reactions. After 7 and 90 days of curing, a decrease in CH of 91% and 54% (Table 6) is observed compared to M0, respectively. Finely ground sand promotes long-term hydration reactions by consuming portlandite from cement hydration [59].

**Figure 5:**
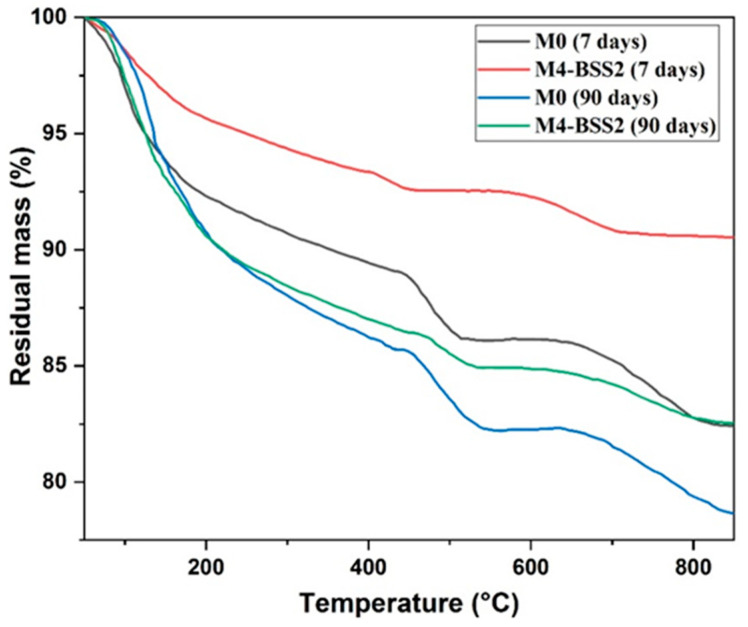
TGA curves of M0 and M4 at 7 and 90 days.

Figure 5 shows the TGA results of M0 and M4 at 7 and 90 days. At an early age (7 days), the mass loss of M0 is higher than that of M4-BSS2. At 90 days, both curves are similar below 450 °C because sand hydration products accelerate the long-term hydration processes.

**Figure 6:**
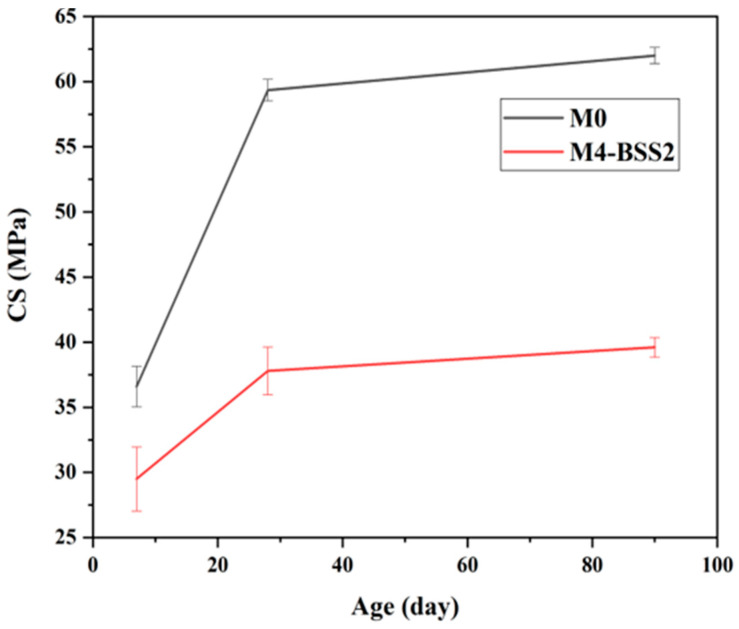
Effect of curing time on CS.

The evolution of compressive strength with age (Figure 6) and the data in Table 6 suggest a correlation between the variation in NEW and CS. Whatever the tested mixture, CS has a decreasing rate over time. CS7 is about 73% of CS90 for M4-BSS2 and 51% of CS90 for M0; CS28 is about 90% of CS90 for both mixtures.

The non-evaporable water content can predict the compressive strength of binary mortars containing finely ground sand. Further studies will confirm the hypothesis and investigate the responsible mechanism.

## 4. Statistical Analysis

The models proposed by Equations (6)–(8) have high correlation coefficients (R^2^ > 0.8 in Table 7) between the predicted and obtained mechanical and physical properties.

Table 8 shows the results of the ANOVA for each modeled response. Since the *t* value of each model is higher than *t_crit_* (*t_crit_* for α = 0.05, p − 1 = 3, and n − p = 8 is equal to 4.07), at least one significant variable is in each model.

Table 9 presents the contribution of each independent variable (i.e., BSS in m^2^/kg and SP in %) and their interaction with SC7, SC28, and WA. In this study, *t_crit_* was 2.306 for n=12 experiments and p = 4 coefficients.

According to Table 9, the independent variables (i.e., SP and BSS) affect CS7, CS28, and WA since |*t*| > *t_crit_*. However, the Student’s *t*-test shows that the interaction between BSS (m^2^/kg) × SP (%) is not significant since |*t*| < *t_crit_*. Therefore, the proposed models can predict the sand powder effects on the mechanical and physical properties of mortars.

**Figure 7:**
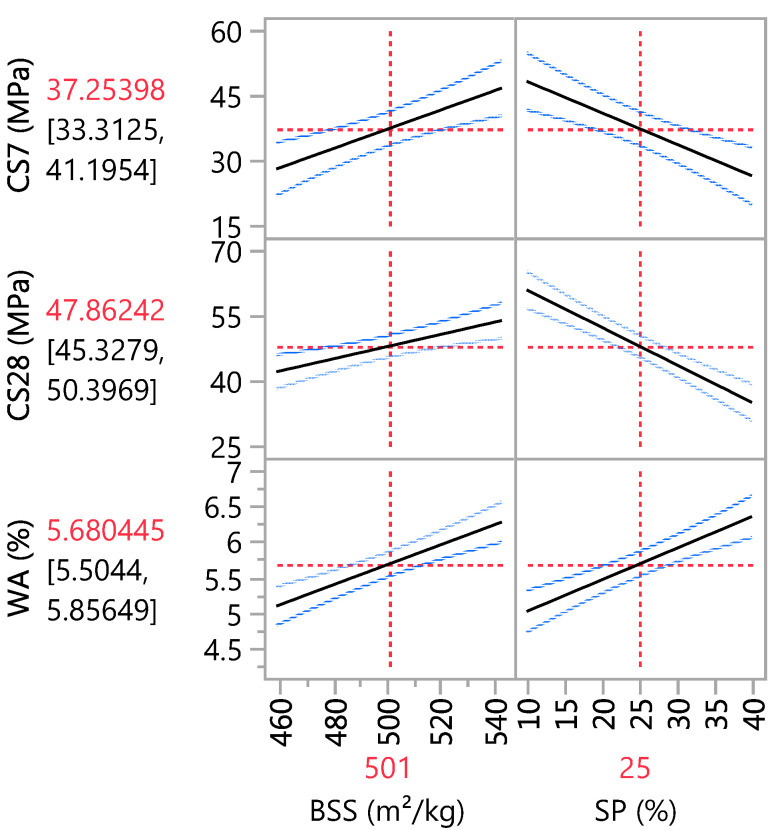
Main effects plot.

The adopted models allow iso-response curves to be drawn based on the independent variables. The main effect plot in Figure 7 confirms that the sand substitution hurts compressive strength, and an increase in SP causes a decrease in CS. On the other hand, BSS has a positive effect on CS. Its influence is more significant at 7 days than at 28 days of curing. This conclusion aligns with the estimation coefficients in Table 9, where the coefficients for BSS and SP are 9.31 and −10.89 at 7-day curing, respectively, and 5.90 and −12.97 at 28-day curing, respectively. Concerning WA, the statistical results indicate that BSS and SP positively affect water absorption. Therefore, an increase in these two variables leads to an increase in water absorption, as in Table 9, where the coefficients for BSS and SP are 0.59 and 0.67, respectively.

Table 10 lists the mechanical properties of the cementitious mortars according to Equation (10).

**Figure 8:**
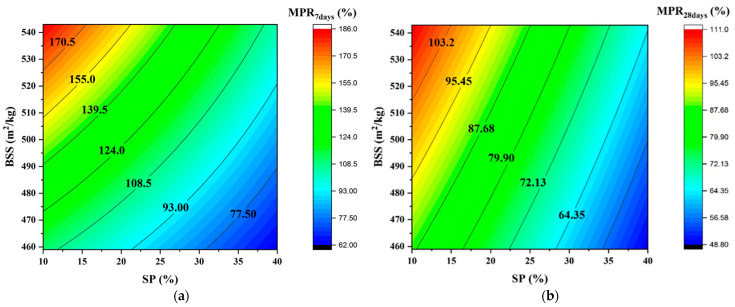
Iso-response curves of MPR. (**a**) at 7 days; (**b**) at 28 days.

Figure 8 depicts the evolution of MPR as a function of SP and BSS and confirms that both significantly affect the mechanical response.

## 5. Conclusions

The environmental burdens of manufacturing are a challenge faced by cement production companies to offer sustainable cement. This study investigates the mechanical and physical properties of binary mortars with finely ground silica sand as a partial substitute for cement. Thirteen mortars have been investigated by varying four substitution ratios from 0% to 40% and three sand fineness values from 459 m^2^/kg to 543 m^2^/kg. Experimental evaluations and statistical data analysis allowed the following conclusions:

- At a young age, the sand BSS does not significantly affect the degree of hydration or amount of portlandite. The increase in sand fineness from 459 to 497 and 543 m^2^/kg decreases the non-evaporable water content from 2.50% to 2.12% and 2.01%, respectively. Moreover, the portlandite content decreased from 4.73% to 4.06% and 4.00% due to the increase in sand fineness.
- Increasing the substitution ratio from 0% to 40% causes a decrease in the non-evaporable water content from 8.65% to 6.22% due to the dilution effect and the portlandite content from 14.55% to 1.19% due to the sand pozzolanic properties.
- The increase in fineness from 459 m^2^/kg to 543 m^2^/kg and the substitution ratio from 0% to 40% led to an increase in water absorption from 4.38% to 6.85% due to the sand’s specific surface area being higher than cement.
- The increase in sand BSS and substitution ratios reduces the volume of cement hydrates and increases porosity and water absorption. Whatever the curing period, the increase in silica sand content causes a decrease in the compressive strength of cement mortars.
- Despite the increase in sand BSS, all mortars maintain water absorption below 10%, ensuring durable and high-performing properties in aggressive environments.
- The statistical analysis of compression and water absorption results demonstrated a strong correlation between the obtained and predicted outcomes, with an R^2^ value exceeding 0.84 and a 95% confidence interval, confirming the validity of the proposed regression models.

Further research should be conducted because this study only included three BSS silica sands, and more binary mixtures should be investigated. Other testing procedures should be conducted to analyze the rheological characteristics of mortars through flow, setting times, shrinkage, microstructure, and durability. Moreover, it is suggested to deepen the micro-mechanisms responsible for water absorption. Finally, the life cycle impacts of binary mortars shall be compared to those of the reference material to assess their environmental effectiveness. This approach will result in better knowledge for practical applications.

## Figures and Tables

**Table 1:** Studies on silica sand as a substitute or mineral addition.

Reference	Used Material	Independent Variables	Main Results
[17]	Desert sand	Cement substitution ratio (0% to 60%)	The desert sand powder in cement hydration was effective for compressive strength between the 28th and 112th days.
[21,27]	Desert sand	Cement substitution ratio (0% to 40%)	The compressive strength decreased with increasing cement substitution by ground desert sand under standard curing. However, it can be a partial cement substitute in precast concrete production.
[28]	Desert sand	BSS (300 and 400 m^2^/kg) Cement substitution ratio (0 to 20%)	The desert sand powder added to Portland cement can reduce CO_2_ emissions and improve the compressive strength and even the consistency of the fresh mixture.
[29]	Desert sand	Cement substitution ratio (0% to 20%)	Desert sand powder improves the workability of fresh mortars.
[30]	Desert sand	Cement substitution ratio (0% to 20%)	Approximately 10% of pozzolan and 10% of dune sand powder ensure optimum resistance. There is a decrease in strength above this percentage.
[31]	Desert sand	Cement substitution ratio (15% to 25%)	Modified mortars made from 20% desert sand are economical and sustainable.
[32]	Quartz	Cement substitution ratio (0% to 20%)	Quartz increases compressive strength and improves the carbonation efficiency of cement mortars.
[33]	Quartz	Cement substitution ratio (0% to 20%)	Quartz reduces the heat of hydration.
[34]	Quartz	BSS (456 and 1232 m^2^/kg)	Quartz contributes to nucleation sites conducive to calcium silicate hydrates in cement mortars.
[35]	Desert and river sand	Crushed sand substitution ratio (0% to 20%)	Binary sands have a positive effect on high-performance self-compacting concrete.

**Table 2:** Chemical analysis of Portland cement and silica sand.

Material (%)	SiO_2_	Al_2_O_3_	Fe_2_O_3_	CaO	MgO	K_2_O	Na_2_O	SO_3_
Cement CEM I 52.5	21.85	4.33	5.22	66.47	-	0.33	-	0.87
Silica sand	98.91	-	0.5	0.52	-	-	-	-

**Table 3:** Main chemical composition of mortars.

Mixture ID	SiO_2_	Al_2_O_3_	Fe_2_O_3_	CaO	K_2_O	SO_3_
M0	21.85	4.33	5.22	66.47	0.33	0.87
M1	29.56	3.90	4.75	59.87	0.30	0.78
M2	37.26	3.46	4.28	53.28	0.26	0.70
M3	44.97	3.03	3.80	46.68	0.23	0.61
M4	52.67	2.60	3.33	40.09	0.20	0.52

**Table 4:** Mixture recipes.

Mixture ID	Crushed Sand (g)	Cement (g)	Sand (g)	Water (g)	Hydraulic Index
M0	-	450	1350	225	0.39
M1	45	405	1350	225	0.55
M2	90	360	1350	225	0.76
M3	135	315	1350	225	1.02
M4	180	270	1350	225	1.37

**Table 5:** Mechanical and physical performances of the mortars.

Mixture ID	7 Days		28 Days		
	CS7 (MPa)	$R_{f}7$ (MPa)	CS28 (MPa)	$R_{f}28$ (MPa)	WA (%)
M0	36.6	5.24	59.35	8.03	4.22
M1-BSS1	30.6	4.97	51.0	6.75	4.38
M2-BSS1	27.6	4.35	43.4	5.49	5.22
M3-BSS1	22.3	3.80	37.7	4.89	5.47
M4-BSS1	16.9	2.92	26.7	3.47	5.70
M1-BSS2	54.9	8.91	65.5	8.67	4.60
M2-BSS2	48.0	7.57	54.5	6.89	5.36
M3-BSS2	39.3	6.70	47.9	6.21	5.80
M4-BSS2	29.5	5.10	37.8	4.91	5.98
M1-BSS3	55.5	9.01	66.1	8.75	5.48
M2-BSS3	48.7	7.68	55.0	6.95	6.33
M3-BSS3	40.1	6.84	48.4	6.27	6.77
M4-BSS3	30.1	5.20	38.1	4.95	6.85

Note: The acronyms of samples refer to the substitution ratio and Blaine fineness of silica sand.

**Table 6:** Effects of curing time on NEW and CH.

Mixture ID	NEW (%)		CH (%)	
	7 Days	90 Days	7 Days	90 Days
M0	8.65	14.89	14.55	17.40
M4-BSS2	6.22	12.20	1.19	8.51

**Table 7:** Summary of fit.

Parameter	CS7	CS28	WA
R^2^	0.84	0.92	0.91
Adjusted R^2^	0.78	0.89	0.88
RMSE	5.92	3.80	0.26
Mean of response	36.96	47.67	5.66

**Table 8:** ANOVA results for the proposed models.

Variable	Source	df	Sum of Squares	Mean Square	*t*
CS7	Model	3	1508.67	502.89	14.36
	Error	8	280.03	35.00	
	Total	11	1788.70	-	
CS28	Model	3	1400.21	466.73	32.24
	Error	8	115.78	14.47	
	Total	11	1516.00	-	
WA	Model	3	5.77	1.92	27.57
	Error	8	0.55	0.07	
	Total	11	6.33	-	

**Table 9:** Effect test.

Variable	Model Term	Estimation	Standard Error	*t*
CS7	Constant	37.25	1.70	21.80
	BSS (m^2^/kg)(459–543)	9.31	2.08	4.46
	SP (%)(10–40)	−10.89	2.29	−4.75
	BSS (m^2^/kg) × SP (%)	−2.77	2.80	−0.99
CS28	Constant	47.86	1.09	43.55
	BSS (m^2^/kg)(459–543)	5.90	1.34	4.40
	SP (%)(10–40)	−12.97	1.47	−8.80
	BSS (m^2^/kg) × SP (%)	−0.87	1.80	−0.48
WA	Constant	5.68	0.07	74.41
	BSS (m^2^/kg)(459–543)	0.59	0.09	6.34
	SP (%)(10–40)	0.66	0.10	6.52
	BSS (m^2^/kg) × SP (%)	0.02	0.12	0.20

**Table 10:** MPR results.

Mixture ID	MPR_7Days_(%)	MPR_28Days_(%)
M0	100%	100%
M1-BSS1	95%	84%
M2-BSS1	85%	70%
M3-BSS1	70%	62%
M4-BSS1	53%	44%
M1-BSS2	170%	108%
M2-BSS2	147%	88%
M3-BSS2	124%	78%
M4-BSS2	93%	62%
M1-BSS3	172%	109%
M2-BSS3	149%	89%
M3-BSS3	126%	79%
M4-BSS3	95%	62%

## Data Availability

The data presented in this study are available on request from the corresponding author.
